# Misalignment in Mechanical Interlocking Heterogeneous Integration: Emergent Behavior and Geometry Optimization

**DOI:** 10.3390/mi16030305

**Published:** 2025-03-04

**Authors:** Matthew Nakamura, Corrisa Heyes, Ethan Rocheville, Kirsten Peterson, Joseph J. Brown

**Affiliations:** Department of Mechanical Engineering, University of Hawai‘i at Mānoa, 2540 Dole Street, Holmes Hall 302, Honolulu, HI 96822, USA; crheyes@hawaii.edu (C.H.); ethanjr@hawaii.edu (E.R.); kp779@hawaii.edu (K.P.); jjbrown@hawaii.edu (J.J.B.)

**Keywords:** mechanical interlocking, heterogeneous integration, cantilever arrays, misalignment sensitivity, snap-through force, nonlinear mechanics, metamaterial surfaces, computational modeling, alignment tolerance, IC device

## Abstract

This paper addresses the challenge of misalignment in cantilever-based mechanical interlocking structures used for the heterogeneous integration of integrated circuits (ICs). As IC applications expand into flexible and multi-functional platforms, precise alignment becomes critical to maintaining optimal mechanical and electrical performance. We investigate the effects of X and Y misalignment on snap-through forces in cantilever arrays, focusing on their impact on mechanical integrity. The experimental results demonstrate that for X-axis misalignments below 15%, the increase in the required snap-through force is less than 5%. In contrast, Y-axis misalignment shows an even more negligible impact, with less than a 5% reduction in force for up to 20% misalignment. Additionally, through polynomial fits of the model across a range of cantilever angles, this study provides a design template for future exploration of cantilever interactions using nonlinear mechanics while minimizing computational load. These findings offer valuable insights for optimizing misalignment tolerance and improving the design of interlocking structures for IC integration, contributing to the development of robust systems for next-generation IC devices.

## 1. Introduction

**Figure 1:**
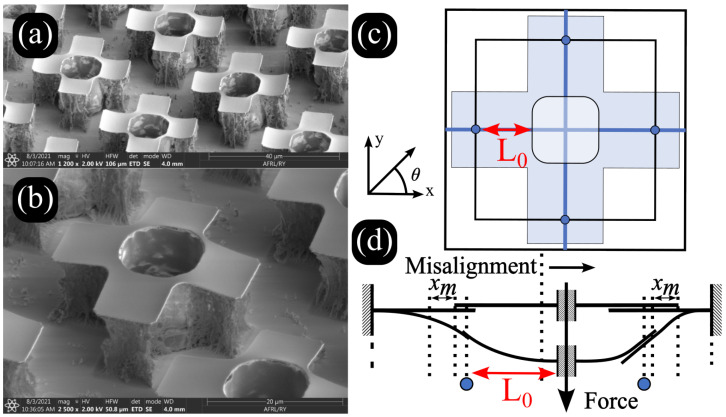
SEM images of mechanically interlocking cantilever structures designed for heterogeneous IC integration. (**a**,**b**) Individual interlocking geometries, showing the intricate cantilever formations essential for mechanical engagement. (**c**) Top-down structure diagram with misalignment variables denoted: $L_{0}$ is the effective cantilever length, and θ is the angle of rotational misalignment with respect to the x,y coordinate plain. (**d**) The change in deflection behavior with misalignment. Misalignment at this scale can lead to mechanical failure or reduced performance, highlighting the need for precision in manufacturing and assembly. SEM Image Credit: Geoffrey Garcia.

The miniaturization of integrated circuits (ICs) and the expansion of their applications into flexible and multi-functional platforms necessitates innovation in material design and assembly techniques [1,2,3]. Heterogeneous integration, which involves the stacking, bonding, power delivery, and signal transmission of IC chips fabricated using different technologies, offers substantial benefits in terms of enhanced functionality and optimized space utilization [4,5,6]. However, this approach also introduces challenges, notably in thermal management, where the increased thermal load in densely packed IC configurations reach temperatures and thermally induced stresses over operational limits [7,8,9]. Recent advancements in metamaterials, specifically those formed through mechanical interlocking structures, present promising pathways for the heterogeneous integration of IC chips across various substrates [10,11,12]. These metamaterial surfaces, composed of tessellated cantilever arrays, offer control over mechanical and electronic properties, enabling the development of next-generation devices with enhanced performance, increased thermal tolerance, and novel functionality [13,14,15]. Examples of these structures can be seen in Figure 1a,b. Previous work has demonstrated the feasibility of mechanically interlocking structures for microelectronic integration, showing their potential to improve interfacial stability and manufacturability while accommodating thermal and mechanical stresses [16]. Mechanically interlocking surfaces enhance mechanical robustness by distributing loads, reducing stress concentrations, and improving fracture toughness, increasing resistance to cyclic loading by up to 50% [17]. Their high locking forces and long-term reliability, demonstrated in active interlocking systems, show potential for stable performance across multiple engagement cycles without significant degradation [18]. Additionally, mechanical contact metasurfaces provide manufacturable, thermally stable alternatives to conventional bonding methods while sustaining mechanical integrity [19]. Furthermore, these structures eliminate solder-related degradation, ensuring reliable electrical contact while reducing parasitic inductance and maintaining stable resistance over extended use [20].

The practical deployment of these complex assemblies in manufacturable systems is significantly dependent on the precision of the alignment of the components [21]. Misalignment in the cantilevers used for mechanical interlocking can lead to suboptimal mechanical properties, reduced reliability, and decreased functional performance, imposing critical limitations on the operational efficiency of the resulting devices [22,23]. Examples of such structure misalignment can be seen in Figure 1c,d. In the context of IC chip integration, the manufacturing reliability of precise electronic responses is paramount; even minor deviations can disrupt the electrical continuity and mechanical stability of the interlocked structure [24,25,26]. Moreover, the scalability of the assembly of such metamaterials poses additional challenges. As the size and complexity of these interlocked systems increase, maintaining uniformity and alignment across large arrays becomes increasingly challenging. This issue is particularly critical in the production of large-area electronic skins and flexible sensors, where uniform mechanical properties are essential for consistent performance.

This paper investigates the effects of misalignment in cantilevers designed for mechanical interlocking, with a focus on their application in creating tiled metamaterial surfaces for heterogeneous integration. We aim to quantify the impact of alignment errors on the mechanical performance of the assemblies, providing information on tolerance thresholds essential to maintain optimal functionality. In addition, this study explores strategies to mitigate the adverse effects of misalignment.

Centimeter-scale experimental cantilever bending data are collected to inform a nonlinear mechanics model for large-deflection beam bending. Through parameter nondimensionalization, these macroscale experimental data may be applied to the behavior of microscale interlocking structures. Prior studies have demonstrated that parameter scaling in physical models effectively predicts mechanical responses across varying sizes, confirming that bulk mechanical behavior follows fundamental physical laws that remain invariant when properly normalized [27]. Although general material properties such as yield strength, fracture toughness, and elastic modulus vary across size scales, the fundamental principles governing snap-through behavior remain geometrically driven and force-dependent, rather than being dominated by material-specific effects [28,29]. Additionally, this study does not rely on assumptions that material properties remain unchanged across scales. Instead, the model is designed to capture force thresholds for snap-through, which are governed by elastic restoring forces that scale predictably with geometry. Research on microscale snap-through instability has shown that geometric nonlinearities, rather than size-dependent material properties, primarily govern behavior, reinforcing the applicability of our experimental findings to the microscale [30]. Furthermore, established experimental methodologies have validated microscale behavior using known scaling techniques, supporting the conclusion that our macroscale results accurately describe the mechanics of microscale structures when valid material properties are applied [31]. Building on this understanding, the next sections will explore how the principles outlined here may be leveraged to develop an analytical model for the effects of array misalignment at the microscale.

## 2. Analytical Model

Misalignment in microscale interlocking structures can arise from manufacturing tolerances, thermal expansion, and dynamic loading, significantly impacting their mechanical behavior and performance. At such small scales, even minor deviations can lead to substantial effects on structural integrity. Therefore, modeling frameworks must incorporate these variations to accurately predict their influence on contact mechanics, force distribution, and overall functionality. The challenge lies in capturing the sensitivity of these structures to misalignment, as small displacements can drastically alter stress distributions, potentially resulting in issues like reduced contact area or failure of the mechanisms.

**Figure 2:**
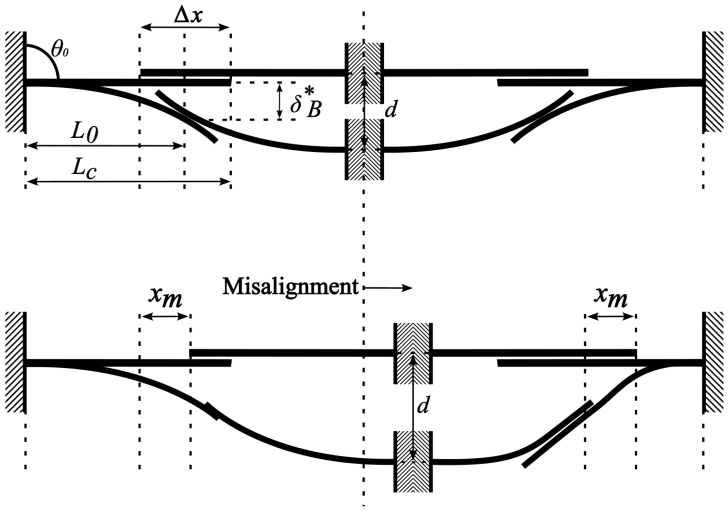
A diagram illustrating aligned (**top**) and in-plane x-direction-misaligned (**bottom**) cantilever systems. The top image shows the interaction length ($L_{0}$) and total cantilever length ($L_{c}$) in the perfectly aligned case, with an overlapping length Δx between the interacting cantilevers. The bottom image depicts the misaligned case, where the offset ($x_{m}$) shifts the interaction point, altering the effective cantilever length and introducing changes in the mechanical response. The $\theta_{0}$ shown indicates the nominal cantilever angle, and $0^{\circ }$ was selected for this work. In both of these diagrams, *d* represents the total downward displacement. The figure is adapted from [32].

To tackle these complexities, a robust modeling framework should include sensitivity analyses to evaluate how misalignment impacts performance. The analytical method utilizes a model based on nonlinear mechanics and large-deflection beam bending theory to account for geometric nonlinearity. The model assumes that the cantilevers behave as continuous, elastic beams with deformation dominated by bending rather than axial stretching or twisting. It neglects friction, adhesion, and out-of-plane bending while considering single-point contact constraints and material homogeneity with linear elastic behavior. These assumptions enable an efficient and accurate representation of interlocking mechanics, ensuring applicability across different length scales while capturing key nonlinear effects of misalignment. A diagram ilistrating the aligned and misaligned cantilever can be seen in Figure 2. Through experiments on cantilever arrays with controlled misalignments, the relationships between force, deflection, and cantilever length are analyzed. The validated model serves as a design template for optimizing cantilever structures, ensuring they can withstand misalignment effects while maintaining reliable mechanical properties for integration in practical applications.

### 2.1. Effective Cantilever Length

We utilize our 1D simplification method [10], where simplification to a 1D model for two interacting cantilevers is possible because the interaction is dominated by bending mechanics along their lengths, while the horizontal (x axis) distance between the cantilever anchors and the contact point remains constant. The model calculates the cantilever’s arc length and curvature up to the contact point, treating the unloaded region beyond as irrelevant. By using the cantilever tip angle as a key variable, the model applies iterative and elliptic integral solutions to predict forces and displacements, enabling efficient analysis of large-deflection mechanics in interlocking systems. The model is reduced to a dependence on the variation in the ‘effective cantilever length’ ($L_{0}$) during interactions between sets of cantilevers. The effective length is critical in determining the mechanical leverage, force distribution, and overall stability of the cantilevered structures. Changes in the effective length due to misalignment can lead to increased mechanical stress, reduced fatigue life, and unexpected failure modes.

Accurate alignment is, therefore, essential for maintaining the intended mechanical properties and ensuring the reliable operation of systems involving cantilevers. Engineers must consider both translational and rotational misalignment during the design and assembly phases to optimize the performance and durability of these systems. This involves the use of precise fabrication techniques, alignment tools, and sometimes active realignment mechanisms in response to operational feedback, which together help mitigate the effects of misalignment and maintain the integrity of cantilever interactions.

### 2.2. Nonlinear Mechanical Behavior

To model the total force required to achieve snap-through for misaligned cantilevers, a modified version of a nonlinear mechanics-based modeling approach from Brown et al. was used [32]. This model uses elliptic integral solutions to calculate the relationships between forces, moments, and deflection at the tips of cantilevers. It takes as inputs definitions for nondimensionalizing the deflection, force, and additional driving parameters to account for generalizing the cantilevers’ specific geometric and load conditions. The model applies transformations of variables and numerical integration to manage the nonlinear deflections observed in cantilevers. This nonlinearity is due to the bending moment in the cantilever beam, which is a function of displacement and the beam’s stiffness, and is initially linear. However, as deflection increases, curvature increases, and the force needed to bend the beam grows faster than in the linear regime [33,34]. This methodology generates output curves that effectively characterize cantilever behavior under different mechanical stresses and enhances predictions of their mechanical responses. This model does not account for the effects of misalignment in the structure arrays.

**Figure 3:**
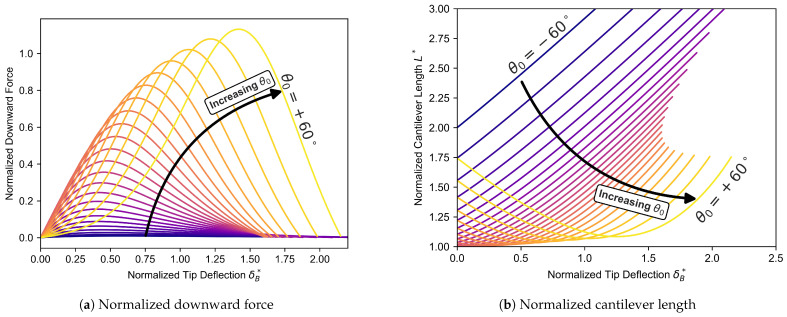
Graphs showing the normalized downward force (**a**) and normalized cantilever length (**b**) as functions of the normalized tip deflection ($\delta_{B}^{*}$) for various initial cantilever angles ($\theta_{0}$). Each curve corresponds to a polynomial fit for a specific angle $\theta_{0}$, ranging from −60° to +60°. These polynomial fits approximate the nonlinear relationships between force, deflection, and cantilever length, reducing the computational load in simulations by providing a simplified mathematical representation of the system’s behavior. The graphs are adapted from [32].

Using a ninth-order polynomial fit to the elliptic integral model results (the coefficients are provided in Appendix A), we simplified the relationship between normalized tip deflection, normalized force, and arc length for the generalized cantilever shown in Figure 3. A ninth-order approximation was chosen as it offered a precise fit to the elliptic solution, with higher-order polynomials yielding no additional improvement in resolution. This polynomial fitting method simplifies the representation of the nonlinear behavior but also enhances the model’s utility for predictive analyses by reducing computational intensity in calculations making use of the model relationships. This model is used to determine the maximum force required for snap-through as a function of the effective length of the cantilever, simulating the effects of misalignment. For the first experiment, to remove one level of complexity, the wall angle for the cantilevers was set to be zero degrees, allowing for the isolation of the translational effects for a given misalignment. For this set, the normalized downward force term is defined as $C_{1}$, and the dimensionless arch length $L^{*}$ as a function of $\delta_{B}^{*}$, the dimensionless cantilever end displacement. The coefficients of the resulting polynomial fits for subsequent initial angles can be found in Appendix A, where fits from −60 to 60 degrees were generated for ease of use.

(1) $$C_{1}=\sum_{n=0}^{9}a_{n}{\left(\delta_{b}^{*}\right)}^{n}$$

(2) $$L^{*}=f\left(\theta_{0},\delta_{B}^{*}\right)$$

Here, the normalized dimensionless cantilever displacement $\delta_{B}^{*}$ is given by the following equation, where *d* is the crosshead displacement, $d_{0}$ is the initial displacement, and $L_{0}$ is the effective cantilever length.

(3) $$\delta_{B}^{*}=\frac{d-d_{0}}{2L_{0}}$$

### 2.3. Misalignment Types

We consider three misalignment types that can occur within the 2D cantilever array: translational in x and y, and rotational. All three directly impact the effective length of the cantilevers and, consequently, the overall mechanical response and stability of the system.

In the case of misalignment in the *X* direction, the effective cantilever length $L_{0}$ for a given misalignment case is given in terms of the initial cantilever length $L_{c}$, the initial overlap Δx, and, in the case of an inline misalignment, the misalignment distance $x_{m}$ as follows:

(4) $$L_{0}=L_{c}-\frac{\Delta x}{2}\pm x_{m}$$

Note that the $x_{m}$ term either causes the effective cantilever length to increase or decrease depending on whether the misalignment is moving closer or further from the fixed point of the cantilever.

To model the effects of the interactions of misaligning the cantilevers in one direction, a set of paired cantilevers is tested. For reference, the cantilevers are broken up into the “Left Set” and the “Right Set”, with the misalignment direction being toward the right. This yields two sets of effective cantilever lengths, $L_{0L}$ and $L_{0R}$:

(5) $$L_{0L}=L_{c}-\frac{\Delta x}{2}+x_{m},\;L_{0R}=L_{c}-\frac{\Delta x}{2}-x_{m}$$

For a y-axis misalignment, we similarly calculate an effective cantilever length (note that $L_{0L}=L_{0R}$ in a pure y-axis shift, so we will denote it as $L_{0y}$ such that given a misalignment distance $y_{m}$, we have:

(6) $$L_{0y}=\sqrt{L_{0}^{2}+y_{m}^{2}}$$

Utilizing the previously described model yields $\delta_{BL}^{*}$ and $\delta_{BR}^{*}$ for the respective normalized dimensionless cantilever displacement, which is used to calculate the normalized downward force terms for each side ($C_{1L}$ and $C_{1R}$). The nondimensional values are redimensioned to make them applicable to true force values as follows:

(7) $$F_{L}=\frac{2EIC_{1L}}{L_{0L}^{2}},\;F_{R}=\frac{2EIC_{1R}}{L_{0R}^{2}},\;\mathrm{and}\;F_{y}=\frac{2EIC_{1R}}{L_{0y}^{2}}$$

Finally, the total insertion force for the interaction can be described as the sum of the left and right sides:

(8) $$P_{x}=F_{L}+F_{R},\;\mathrm{or}\;P_{y}=2F_{y}$$

Due to the variation in the effective interaction length caused by the offset, each side will snap through at different displacement distances. To understand this phenomenon, these curves can be plotted by using $\delta_{BL}^{*}$ and $\delta_{BR}^{*}$ with the dimensionless arch length $L^{*}$, yielding $L_{L}^{*}$ and $L_{R}^{*}$, which can be redimensioned as follows:

(9) $$L_{L}=L_{L}^{*}+L_{0L},\;\mathrm{and}\;L_{R}=L_{R}^{*}+L_{0R}$$

**Figure 4:**
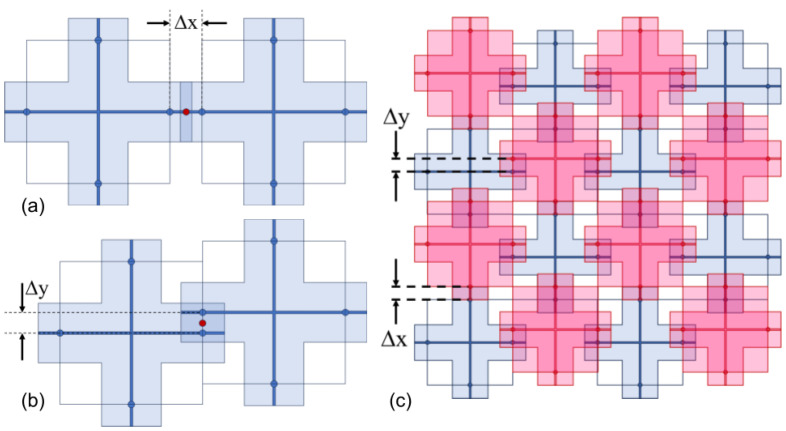
Illustration of translational misalignments. (**a**) The relocated centroid of cantilever overlap in an x-shift misalignment. (**b**) The relocation of the centroid in a y-shift misalignment. (**c**) The translational misalignment of a vertical shift with red cantilevers offset relative to blue cantilevers, leading to variations in the effective interaction length and changes in mechanical performance. Note the misalignment in both the x and y directions.

In-plane translational misalignment in Y occurs when cantilevers are displaced from their intended positions while maintaining proper orientation. This type of misalignment results in a shift in the cantilever’s position relative to its intended point of contact. The primary consequence of translational misalignment is variation in the overlap or engagement length between interacting cantilevers, as can be seen in Figure 4. Such shifts can lead to uneven stress distributions and localized points of high strain, potentially compromising the structural integrity and functional accuracy of the assembly.

In-plane rotational misalignment involves the angular displacement of a cantilever array around a point in the array. This misalignment can result in angular mismatch between interacting cantilevers, leading to improper force distribution at the interaction points. The deviation in angle can alter the effective length of engagement between cantilevers, introducing a torque to the structures, thus affecting the load distribution and mechanical efficiency of the system.

**Figure 5:**
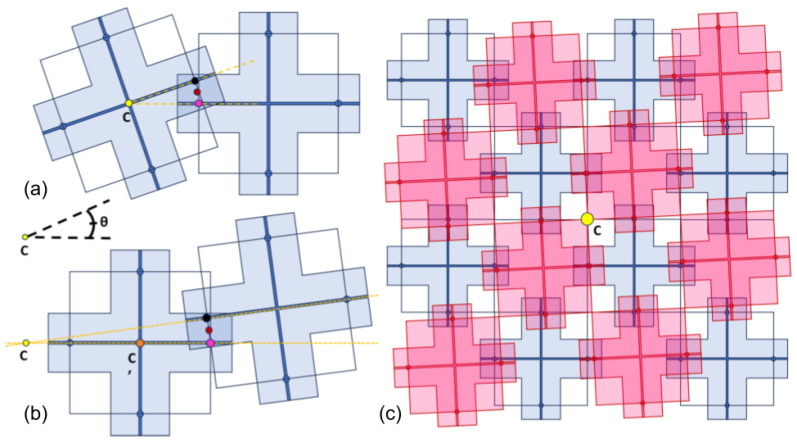
A rotation misalignment may be (**a**) centered around a structure, which is a special case, or (**b**) NOT centered around the structure. In either case, the new centroid (red) is at the midpoint between previously overlapping points (black and pink) of the cantilever leaf. (**c**) A top-down view of an array of multiple structures, where the blue array is approached by a rotationally misaligned red array (rotation centered at point in yellow).

For rotational misalignments centered on a structure, as shown in Figure 5a, we may calculate the sets of effective cantilever lengths as the geometric mean of the separation from the ideal overlap. That is, given points $\left(x_{1},y_{1}\right)$ in pink and $\left(x_{2},y_{2}\right)=\left(x_{1}\cos\theta -y_{1}\sin\theta ,x_{1}\sin\theta +y_{1}\cos\theta \right)$ in black with our origin at the center of rotation in yellow $C=\left(x_{c},y_{c}\right)=\left(0,0\right)$, we define the coordinates of the midpoint $M=(x_{m},y_{m})$ in red to be

(10) $$x_{m}=L_{0}=0.5\left(x_{1}-x_{2}\right),\;y_{m}=0.5\left(y_{1}-y_{2}\right)$$

And we can then determine the rotational effective cantilever lengths:

(11) $$RotL_{0L}=\left|M\right|=\sqrt{{\left(L_{0}-0.5\left(x_{1}-x_{2}\right)\right)}^{2}+{\left(0.5\left(y_{1}-y_{2}\right)\right)}^{2}}$$

Furthermore, due to rotational symmetry, we have

(12) $$RotL_{0R}=\sqrt{{\left(L_{0}+0.5\left(x_{1}-x_{2}\right)\right)}^{2}+{\left(0.5\left(y_{1}-y_{2}\right)\right)}^{2}}$$

For a rotation not centered around one of the structures of consideration (shown in Figure 5b), the calculation becomes more complicated. First we define our center of rotation $C=(x_{c},y_{c})$, shown in yellow, and the point $C^{\prime }=\left(x_{c}^{\prime },y_{c}^{\prime }\right)$ in orange at the center of the structure so that we may consider $L_{0}$, the distance between $C^{\prime }$, and $\left(x_{1},y_{1}\right)$ in pink as before. Then, we locate the black point $\left(x_{2},y_{2}\right)$ with respect to the origin at *C* by translating $L_{0}$ to the origin of our rotation and performing a rotation with θ and moving it back, such that:

(13) $$x_{2}=\left(x_{1}-x_{c}^{\prime }\right)\cos\left(\theta \right)-\left(y_{1}-y_{c}^{\prime }\right)\sin\left(\theta \right)+x_{c}^{\prime }$$

(14) $$y_{2}=\left(x_{1}-x_{c}^{\prime }\right)\sin\left(\theta \right)+\left(y_{1}-y_{c}^{\prime }\right)\cos\left(\theta \right)+y_{c}^{\prime }$$

Then, we may perform the same calculations to find the geometric mean between points $\left(x_{1},y_{1}\right)$ and $\left(x_{2},y_{2}\right)$, as shown in Equation (10). Furthermore, we have the same rotational symmetry here because of the way that $\left(x_{1},y_{1}\right)$ and $\left(x_{2},y_{2}\right)$ are defined with respect to their respective structure centers.

The analytical model presented here offers a method for quantifying the maximum force required for cantilever snap-through as a function of translational offsets in the *x* and *y* directions, as well as rotational misalignments. By incorporating geometric nonlinearity and sensitivity to misalignment, this model provides a framework for evaluating the mechanical response under varying conditions, enabling the prediction of system behavior and the identification of critical thresholds that may impact structural performance.

## 3. Physical Experiment

Verification and validation of the models through experimental data were performed to validate our findings and ensure model accuracy.

### 3.1. Experimental Setup and Procedure for Cantilevers in Perfect Alignment

This study aimed to determine the impact of misalignment on the push-through force of cantilevers of even lengths. The testing was conducted using a Shimadzu AGS-X series mechanical testing machine in the compression configuration. The cantilevers had dimensions of 14.7±0.2 mm in width, 0.6 mm in height, and 32±0.2 mm in length. The cantilevers were tested using a custom 3D-printed PLA jig. The bottom jig was bolted into the Shimadzu machine, while the top jig was slid onto the crosshead and secured with a bolt. The bottom jig was able to translate left and right to achieve the desired offset needed for the tests. The cantilevers were loaded into the bottom jig by sliding them in and tightening the screws to clamp them down. The alignment was then checked by bringing the gantry down until the cantilevers touched. After this, the force and displacement readings on the Shimadzu machine were zeroed and the test could begin.

The first trial began with an even overlap of approximately 7 mm on both sides of the cantilevers. The Shimadzu machine was programmed using Trapezium-X software to push down at a constant rate of 1 mm/s until both sides pushed through. The machine would then stop automatically. After each test, the force and displacement data were saved as a comma-separated-variable file. The machine was then reset for the next test and the setup procedure was repeated with a minimum of 5 times per trial.

### 3.2. Experimental Setup and Procedure for Translationally Misaligned Cantilevers

**Figure 6:**
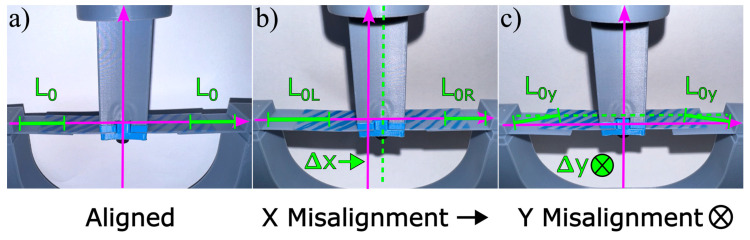
Shown above is the experimental apparatus loaded into the Shimadzu AGS-X series mechanical testing machine, where the cantilevers with blue crosshatching are the cantilevers moving into contact with the solid colored cantilevers. (**a**) The perfect alignment case where there is equal overlap with no misalignment. (**b**) The case where there is a misalignment in the x direction, where $L_{0L}$ is larger than $L_{0R}$. (**c**) The case where there is a misalignment in the y direction, yielding an increase equal to the effective length, denoted by $L_{0y}$.

After completing a trial, the cantilevers were replaced with a new set, and the bottom jig was shifted 1 mm to the left. To avoid any potential fatigue in the samples, the cantilevers were replaced after each run. This process was repeated until there was an 8 mm offset on the left side, resulting in a total of 10 trials. The configuration of the interacting cantilevers, as shown in Figure 6, illustrates the experimental setup within the Shimadzu AGS-X series mechanical testing machine. The figure highlights the cantilevers with blue crosshatching moving into contact with the solid-colored cantilevers and demonstrates variations in alignment, including perfect alignment and cases of misalignment in both the x and y directions, affecting the overlap and effective lengths of the cantilevers. The stages of a misalignment interaction between two sets of cantilevers, including the early snap-through on one side, are shown in Appendix B, where initially, the left side of the cantilever extends to its full length and achieves the first snap-through. Subsequently, the cantilever progresses to the right, reaching a secondary snap-through point at a further distance, as described by the analytical model.

## 4. Results and Discussion

**Figure 7:**
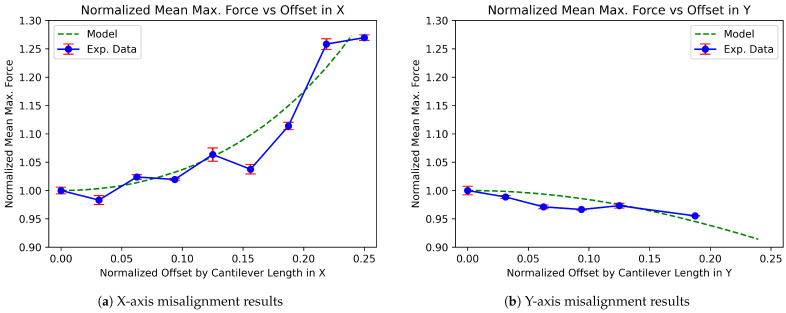
The results from the misalignment experiments performed for X- and Y-direction misalignment (left and right, respectively). Note the magnified scale to illustrate small deviations from the model. (**a**) X-axis misalignment. (**b**) Y-axis misalignment.

The data and code used in this study are publicly available. The dataset can be accessed on Zenodo at [35], and the codebase is hosted on GitHub and archived on Zenodo at [36]. These resources are provided to ensure transparency and reproducibility of the results presented in this work. The graphs presented in Figure 7 show the results from the X and Y misalignment experiments. In each, the experimentally collected data with standard deviation bars are shown in blue, and the predicted force output of the model is given in green. In the case of the X misalignment, the experimental data closely follow the trend to the model’s predicted force and highlight that for small numbers of in-line misalignment (less than 15%), there is minimal change in the overall load required to have the cantilever snap-through (less than 5% more force). The overall agreement between the experimental results and the model indicates that 2D translational misalignment has a notable impact on the mechanical force as the offset increases, leading to a more pronounced rise in the required force for snap-through.

In contrast, for the Y misalignment, both the model and experimental data indicate a much smaller effect on the normalized mean maximum force as the offset increases. The experimental data show a slight decline, but the magnitude of change is minimal (less than a 5% reduction in force with a ≈20% offset), suggesting that changes in the effective cantilever length due to translation misalignment in the y direction do not significantly affect the force required to achieve snap-through.

Rotational misalignment, as a combination of both X- and Y-offsets, presents a more complex interaction in determining the overall mechanical behavior of cantilever arrays. Based on these results, the data suggest that the force required for snap-through in an array of rotated cantilevers is primarily governed by the X component of misalignment, with the Y component contributing significantly less to the total force. However, it is essential to note that there is a degree of tolerance allowed in the X-offset due to the nonlinear relationship between the force and changes in the effective cantilever length. At small X-offsets (less than 15%), the increase in force required remains relatively minimal, even as the effective length changes. This nonlinear behavior means that slight variations in X translation do not lead to a proportional increase in force, providing some tolerance in the alignment process. Beyond this threshold, the force increases more sharply, emphasizing the importance of control over X-axis alignment within these tolerances.

This highlights that in the design and assembly of mechanically interlocking structures, controlling the X-axis alignment remains critical, even when rotational misalignment is considered. While there is some tolerance due to the nonlinear force–length relationship, large deviations in X translation will significantly affect the mechanical response. Meanwhile, the Y-axis offset can be tolerated to a greater degree without drastically affecting performance, but precision in the X direction is essential for maintaining mechanical integrity and ensuring consistent force responses across the array.

## 5. Conclusions

This study reproduced and expanded upon a large-deflection beam bending model to account for in-plane translational and rotational misalignments in mechanical interlocking cantilever arrays. By validating the model through physical experiments, we demonstrated that X-axis misalignment significantly influences the snap-through force, while Y-axis misalignment has a much smaller impact. These results show that for X-axis misalignments below 15%, the increase in required snap-through force is less than 5%, and Y-axis misalignment causes less than a 5% reduction in force even at a 20% offset. The nonlinear relationship between force and practical cantilever length introduces a degree of tolerance for small X-offsets, allowing for some variation in alignment without drastically affecting performance. However, more significant deviations in X misalignment result in sharp increases in the required force, with the force rising steeply beyond the 15% threshold. This result underscores the availability of tolerance during the assembly of MIS structures.

**Figure 8:**
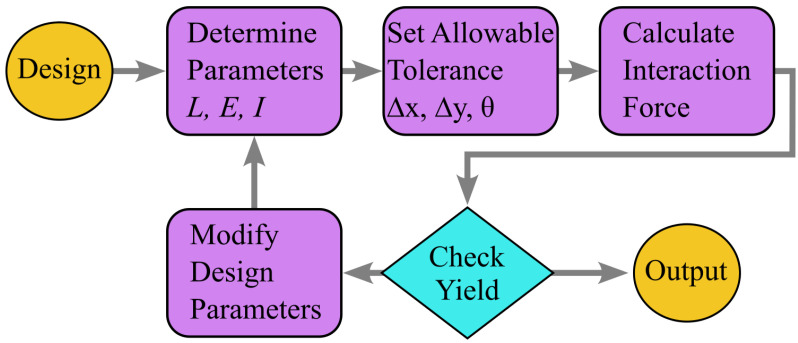
Future MIS designs with cantilevers will start by defining geometric and material parameters (*L*, *E*, *I*), then setting allowable tolerances (Δx, Δy, θ). Interaction forces will be calculated, and yield will be evaluated to refine the parameters, ensuring an optimal design through this framework.

Moreover, the use of polynomial fits to model cantilever interactions across a range of angles offers an efficient design template for future exploration of nonlinear mechanical behaviors in interlocking structures. These fits simplify the complex interactions while minimizing computational load, enabling further optimization of cantilever arrays without compromising accuracy. Future designs should utilize the workflow illustrated in Figure 8 to establish geometric parameters, tolerances, and evaluation criteria effectively. This framework provides a pathway for future research into various cantilever geometries and interactions while balancing model complexity and computational efficiency. In conclusion, controlling X-axis alignment remains critical but has a tolerant region in maintaining the mechanical performance of these systems, while Y-axis misalignment plays a secondary role. Future work may explore additional misalignment scenarios and optimize designs to further improve the robustness and functionality of interlocking systems for IC integration.

## Figures and Tables

**Figure A1:**
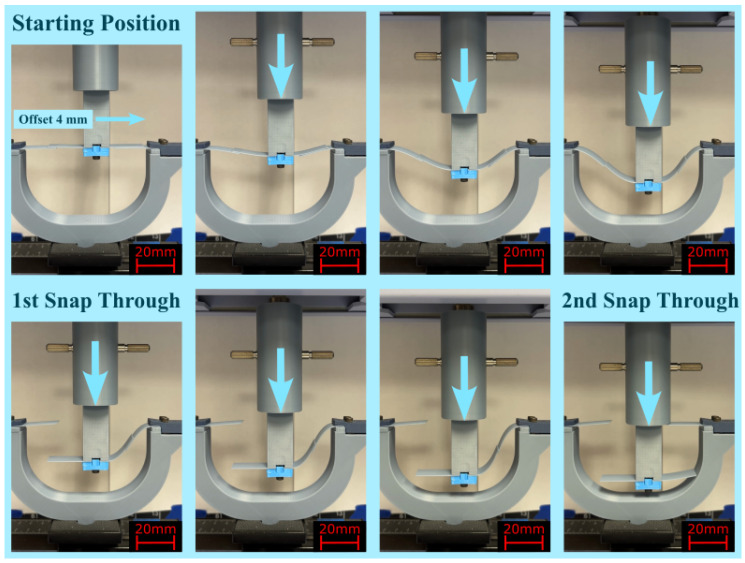
This figure presents a series of eight snapshots from an x misalignment experiment, 4 mm to the right. Initially, the left side of the cantilever extends to its full length and achieves the first snap-through. Subsequently, the cantilever progresses to the right, reaching a secondary snap-through point at a further distance, as described by the analytical model.

## Data Availability

The codebase supporting this study is openly available on GitHub at https://github.com/nanosystemslab/Cantilever_Misalignment_Interaction and is archived on Zenodo at https://doi.org/10.5281/zenodo.14679787. The dataset used in this research is hosted on Zenodo and is available at https://doi.org/10.5281/zenodo.14654525. These resources are provided to ensure transparency and reproducibility of the results (last accessed 3 February 2025).

## Abbreviations

The following abbreviations are used in this manuscript:

$L_{0}$	Effective cantilever length
$L_{0L}$	Left set effective cantilever length
$L_{0R}$	Right set effective cantilever length
$L_{0y}$	y-axis shift effective cantilever length
$L_{c}$	Overlap length
Δx	Cantilever overlap in x direction
$x_{m}$	Misalignment offset in x direction
$y_{m}$	Misalignment offset in y direction
*M*	Misalignment midpoint $\left(x_{m},y_{m}\right)$
*C*	Rotational misalignment center $\left(x_{c},y_{c}\right)$
$C^{\prime }$	Structure center
θ	Angle of rotational misalignment
$\delta_{B}^{*}$	Normalized tip deflection
$\delta_{BR}^{*}$	Right set normalized tip deflection
$\delta_{BL}^{*}$	Left set normalized tip deflection
$\theta_{0}$	Initial cantilever angle
$C_{1}$	Normalized downward force
$C_{1R}$	Right set normalized downward force
$C_{1L}$	Left set normalized downward force
$L^{*}$	Dimensionless arch length
*d*	Crosshead displacement
$d_{0}$	Initial displacement
Δy	Cantilever overlap in y direction
$y_{m}$	Misalignment offset in y direction
$P_{x}$	Total insertion force given x misalignment
$P_{y}$	Total insertion force given y misalignment
$F_{L}$	Left set insertion force
$F_{R}$	Right set insertion force
$F_{y}$	One-sided y misalignment insertion force
$RotL_{0L}$	Left set rotational effective cantilever length
$RotL_{0R}$	Right set rotational effective cantilever length

## Appendix A. Coefficients of Polynomial Fits

### Appendix A.1. Coefficients of Polynomial Fits for Normalized Downward Force

**Table A1:** This table presents the coefficients of polynomial fits for normalized downward force, showcasing how these coefficients vary across a range of angles from −60 to 60 degrees, in increments of 5 degrees. Each entry includes coefficients from X9 to C (constant term), adjusting to the changes in the angle. The ‘Delta B Limits’ columns indicate the minimum and maximum boundaries that denote the points of snap-through.

	Coefficients of Polynomial Fits for Normalized Downward Force										Delta B Limits	
Angle (°)	$X^{9}$	$X^{8}$	$X^{7}$	$X^{6}$	$X^{5}$	$X^{4}$	$X^{3}$	$X^{2}$	$X^{1}$	C	Min.	Max.
**−60**	$1.06633099\times 10^{-05}$	$-2.10078894\times 10^{-04}$	$1.80570481\times 10^{-03}$	$-8.94107852\times 10^{-03}$	$2.84155362\times 10^{-02}$	$-6.11292877\times 10^{-02}$	$9.06999867\times 10^{-02}$	$-8.93246850\times 10^{-02}$	$4.66408059\times 10^{-02}$	$6.06572949\times 10^{-06}$	0.0	3.75605
**−55**	$4.45391353\times 10^{-05}$	$-7.83641835\times 10^{-04}$	$6.05302962\times 10^{-03}$	$-2.71436112\times 10^{-02}$	$7.88194639\times 10^{-02}$	$-1.56238222\times 10^{-01}$	$2.14617891\times 10^{-01}$	$-1.95113157\times 10^{-01}$	$9.29223349\times 10^{-02}$	$4.80233800\times 10^{-06}$	0.0	3.27224
**−50**	$1.05165859\times 10^{-04}$	$-1.75614870\times 10^{-03}$	$1.30026582\times 10^{-02}$	$-5.64602291\times 10^{-02}$	$1.60128173\times 10^{-01}$	$-3.11316862\times 10^{-01}$	$4.17893566\times 10^{-01}$	$-3.66315161\times 10^{-01}$	$1.64692129\times 10^{-01}$	$-1.07313997\times 10^{-06}$	0.0	2.91031
**−45**	$2.55015118\times 10^{-05}$	$-1.03730569\times 10^{-03}$	$1.17028129\times 10^{-02}$	$-6.58796979\times 10^{-02}$	$2.21632680\times 10^{-01}$	$-4.80231175\times 10^{-01}$	$6.82827644\times 10^{-01}$	$-6.06535147\times 10^{-01}$	$2.65895441\times 10^{-01}$	$-1.21182559\times 10^{-05}$	0.0	2.63118
**−40**	$-9.22760541\times 10^{-04}$	$9.49392223\times 10^{-03}$	$-3.61178792\times 10^{-02}$	$4.38104130\times 10^{-02}$	$1.09521683\times 10^{-01}$	$-5.19144640\times 10^{-01}$	$9.33995687\times 10^{-01}$	$-8.99465983\times 10^{-01}$	$3.97327751\times 10^{-01}$	$-2.61952729\times 10^{-05}$	0.0	2.41101
**−35**	$-4.49988933\times 10^{-03}$	$4.77336924\times 10^{-02}$	$-2.07916390\times 10^{-01}$	$4.59420402\times 10^{-01}$	$-4.51673306\times 10^{-01}$	$-1.73565064\times 10^{-01}$	$1.02956162\times 10^{+00}$	$-1.20443005\times 10^{+00}$	$5.55679731\times 10^{-01}$	$-3.77712187\times 10^{-05}$	0.0	2.23450
**−30**	$-1.31644369\times 10^{-02}$	$1.36653112\times 10^{-01}$	$-5.96447645\times 10^{-01}$	$1.39498729\times 10^{+00}$	$-1.77529798\times 10^{+00}$	$8.57566066\times 10^{-01}$	$7.85707234\times 10^{-01}$	$-1.45931140\times 10^{+00}$	$7.33229349\times 10^{-01}$	$-3.86229347\times 10^{-05}$	0.0	2.09137
**−25**	$-2.72992549\times 10^{-02}$	$2.78656797\times 10^{-01}$	$-1.21162619\times 10^{+00}$	$2.88974129\times 10^{+00}$	$-3.96982342\times 10^{+00}$	$2.74997779\times 10^{+00}$	$4.45011742\times 10^{-02}$	$-1.59300655\times 10^{+00}$	$9.18374756\times 10^{-01}$	$-2.05000727\times 10^{-05}$	0.0	1.97454
**−20**	$-3.90085971\times 10^{-02}$	$4.04587134\times 10^{-01}$	$-1.80477692\times 10^{+00}$	$4.47697744\times 10^{+00}$	$-6.56624758\times 10^{+00}$	$5.29853990\times 10^{+00}$	$-1.22331941\times 10^{+00}$	$-1.54645005\times 10^{+00}$	$1.09703084\times 10^{+00}$	$2.03152027\times 10^{-05}$	0.0	1.87897
**−15**	$-2.52441894\times 10^{-02}$	$3.22676967\times 10^{-01}$	$-1.70740614\times 10^{+00}$	$4.89784341\times 10^{+00}$	$-8.20068843\times 10^{+00}$	$7.68953376\times 10^{+00}$	$-2.81643651\times 10^{+00}$	$-1.29705283\times 10^{+00}$	$1.25468064\times 10^{+00}$	$7.76356341\times 10^{-05}$	0.0	1.80106
**−10**	$5.06958334\times 10^{-02}$	$-2.64376634\times 10^{-01}$	$1.03468649\times 10^{-01}$	$2.21325953\times 10^{+00}$	$-6.69388436\times 10^{+00}$	$8.49460703\times 10^{+00}$	$-4.26323489\times 10^{+00}$	$-8.77236611\times 10^{-01}$	$1.37862383\times 10^{+00}$	$1.31534412\times 10^{-04}$	0.0	1.73820
**−5**	$2.17106939\times 10^{-01}$	$-1.59047933\times 10^{+00}$	$4.47257036\times 10^{+00}$	$-5.30710204\times 10^{+00}$	$1.15801000\times 10^{-01}$	$6.09088145\times 10^{+00}$	$-4.91253066\times 10^{+00}$	$-3.75643324\times 10^{-01}$	$1.45977619\times 10^{+00}$	$1.49478455\times 10^{-04}$	0.0	1.68851
**0**	$4.48828445\times 10^{-01}$	$-3.49398239\times 10^{+00}$	$1.10416293\times 10^{+01}$	$-1.74756858\times 10^{+01}$	$1.27188285\times 10^{+01}$	$-4.24339607\times 10^{-01}$	$-4.19196275\times 10^{+00}$	$8.80033019\times 10^{-02}$	$1.49335446\times 10^{+00}$	$9.59742904\times 10^{-05}$	0.0	1.65069
**5**	$6.24152257\times 10^{-01}$	$-5.09535729\times 10^{+00}$	$1.72213431\times 10^{+01}$	$-3.03768473\times 10^{+01}$	$2.80424628\times 10^{+01}$	$-1.00710320\times 10^{+01}$	$-1.99051707\times 10^{+00}$	$4.14440514\times 10^{-01}$	$1.47805410\times 10^{+00}$	$-4.76592177\times 10^{-05}$	0.0	1.62393
**10**	$5.38805074\times 10^{-01}$	$-4.85949795\times 10^{+00}$	$1.82169498\times 10^{+01}$	$-3.60475860\times 10^{+01}$	$3.86899388\times 10^{+01}$	$-1.92385389\times 10^{+01}$	$9.97724499\times 10^{-01}$	$5.87206920\times 10^{-01}$	$1.41397387\times 10^{+00}$	$-2.56847677\times 10^{-04}$	0.0	1.60779
**15**	$2.61151046\times 10^{-02}$	$-1.39714497\times 10^{+00}$	$9.18497811\times 10^{+00}$	$-2.54258846\times 10^{+01}$	$3.50112353\times 10^{+01}$	$-2.22862474\times 10^{+01}$	$3.23935674\times 10^{+00}$	$7.11791915\times 10^{-01}$	$1.30051032\times 10^{+00}$	$-4.42621983\times 10^{-04}$	0.0	1.60228
**20**	$-8.46220730\times 10^{-01}$	$5.12222383\times 10^{+00}$	$-1.05852387\times 10^{+01}$	$5.12543551\times 10^{+00}$	$1.07685475\times 10^{+01}$	$-1.41691040\times 10^{+01}$	$2.90893704\times 10^{+00}$	$9.86691677\times 10^{-01}$	$1.13640528\times 10^{+00}$	$-4.57686200\times 10^{-04}$	0.0	1.60779
**25**	$-1.65911027\times 10^{+00}$	$1.19229291\times 10^{+01}$	$-3.38994260\times 10^{+01}$	$4.67488689\times 10^{+01}$	$-2.94874888\times 10^{+01}$	$5.35768810\times 10^{+00}$	$-9.30852632\times 10^{-01}$	$1.57456390\times 10^{+00}$	$9.24322610\times 10^{-01}$	$-1.56248549\times 10^{-04}$	0.0	1.62521
**30**	$-1.81047869\times 10^{+00}$	$1.44194558\times 10^{+01}$	$-4.66043614\times 10^{+01}$	$7.69793126\times 10^{+01}$	$-6.66905933\times 10^{+01}$	$2.82702321\times 10^{+01}$	$-6.96311484\times 10^{+00}$	$2.39622612\times 10^{+00}$	$6.80566733\times 10^{-01}$	$4.79759050\times 10^{-04}$	0.0	1.65602
**35**	$-9.92777366\times 10^{-01}$	$9.43126479\times 10^{+00}$	$-3.60960831\times 10^{+01}$	$7.07149141\times 10^{+01}$	$-7.39299313\times 10^{+01}$	$3.94158600\times 10^{+01}$	$-1.11398809\times 10^{+01}$	$2.96854520\times 10^{+00}$	$4.45676749\times 10^{-01}$	$1.18575547\times 10^{-03}$	0.0	1.70254
**40**	$3.78100057\times 10^{-01}$	$-1.12992138\times 10^{+00}$	$-3.74802380\times 10^{+00}$	$2.13120065\times 10^{+01}$	$-3.54812455\times 10^{+01}$	$2.52459850\times 10^{+01}$	$-8.42183511\times 10^{+00}$	$2.50702071\times 10^{+00}$	$2.83997610\times 10^{-01}$	$1.36259511\times 10^{-03}$	0.0	1.76827
**45**	$1.33516968\times 10^{+00}$	$-1.01627008\times 10^{+01}$	$3.02560086\times 10^{+01}$	$-4.39648555\times 10^{+01}$	$3.21911786\times 10^{+01}$	$-1.22954457\times 10^{+01}$	$2.89648801\times 10^{+00}$	$4.96378505\times 10^{-01}$	$2.54750844\times 10^{-01}$	$3.53308604\times 10^{-04}$	0.0	1.85846
**50**	$1.21965381\times 10^{+00}$	$-1.10315209\times 10^{+01}$	$4.03650907\times 10^{+01}$	$-7.63994628\times 10^{+01}$	$8.02172307\times 10^{+01}$	$-4.80535058\times 10^{+01}$	$1.66470636\times 10^{+01}$	$-2.38373288\times 10^{+00}$	$3.46189165\times 10^{-01}$	$-1.79423414\times 10^{-03}$	0.0	1.98128
**55**	$3.88904000\times 10^{-01}$	$-4.50772672\times 10^{+00}$	$2.08160346\times 10^{+01}$	$-4.93122173\times 10^{+01}$	$6.45104852\times 10^{+01}$	$-4.75812458\times 10^{+01}$	$1.97424984\times 10^{+01}$	$-3.78415021\times 10^{+00}$	$4.06251744\times 10^{-01}$	$-3.44918128\times 10^{-03}$	0.0	2.14976
**60**	$-2.1707657\times 10^{-01}$	$1.79139318\times 10^{+00}$	$-5.40851112\times 10^{+00}$	$6.81466434\times 10^{+00}$	$-1.87877466\times 10^{+00}$	$-3.46294659\times 10^{+00}$	$3.62908236\times 10^{+00}$	$-1.08426567\times 10^{+00}$	$1.78410689\times 10^{-01}$	$-1.85948835\times 10^{-03}$	0.0	2.38593

### Appendix A.2. Coefficients of Polynomial Fits for Normalized Cantilever Length

**Table A2:** This table presents the coefficients of polynomial fits for normalized cantilever lengths, showcasing how these coefficients vary across a range of angles from −60 to 60 degrees, in increments of 5 degrees. Each entry includes coefficients from X9 to C (constant term), adjusting to the changes in the angle. The ‘Delta B Limits’ columns indicate the minimum and maximum boundaries that denote the points of snap-through.

	Coefficients of Polynomial Fits for Normalized Cantilever Length										Delta B Limits	
Angle (°)	$X^{9}$	$X^{8}$	$X^{7}$	$X^{6}$	$X^{5}$	$X^{4}$	$X^{3}$	$X^{2}$	$X^{1}$	C	Min.	Max.
**−60**	$-8.687388\times 10^{-07}$	$1.828882\times 10^{-05}$	$-1.733267\times 10^{-04}$	$9.957697\times 10^{-04}$	$-3.989313\times 10^{-03}$	$1.233629\times 10^{-02}$	$-3.208978\times 10^{-02}$	$7.489104\times 10^{-02}$	$8.660383\times 10^{-01}$	$2.000000\times 10^{+00}$	0.0	$3.756058\times 10^{+00}$
**−55**	$-2.356786\times 10^{-06}$	$4.467114\times 10^{-05}$	$-3.847804\times 10^{-04}$	$2.031866\times 10^{-03}$	$-7.564576\times 10^{-03}$	$2.186712\times 10^{-02}$	$-5.292828\times 10^{-02}$	$1.131491\times 10^{-01}$	$8.191598\times 10^{-01}$	$1.743447\times 10^{+00}$	0.0	$3.272247\times 10^{+00}$
**−50**	$-3.844015\times 10^{-06}$	$7.059483\times 10^{-05}$	$-5.969339\times 10^{-04}$	$3.129505\times 10^{-03}$	$-1.162741\times 10^{-02}$	$3.334747\times 10^{-02}$	$-7.860883\times 10^{-02}$	$1.593704\times 10^{-01}$	$7.660434\times 10^{-01}$	$1.555724\times 10^{+00}$	0.0	$2.910315\times 10^{+00}$
**−45**	$-5.987557\times 10^{-07}$	$3.505562\times 10^{-05}$	$-4.735611\times 10^{-04}$	$3.246895\times 10^{-03}$	$-1.416227\times 10^{-02}$	$4.420812\times 10^{-02}$	$-1.069109\times 10^{-01}$	$2.122845\times 10^{-01}$	$7.070950\times 10^{-01}$	$1.414214\times 10^{+00}$	0.0	$2.631180\times 10^{+00}$
**−40**	$1.906689\times 10^{-05}$	$-1.941536\times 10^{-04}$	$6.301129\times 10^{-04}$	$5.876003\times 10^{-04}$	$-1.195870\times 10^{-02}$	$5.052324\times 10^{-02}$	$-1.344646\times 10^{-01}$	$2.700099\times 10^{-01}$	$6.427662\times 10^{-01}$	$1.305408\times 10^{+00}$	0.0	$2.411016\times 10^{+00}$
**−35**	$7.282771\times 10^{-05}$	$-7.963512\times 10^{-04}$	$3.509588\times 10^{-03}$	$-6.920522\times 10^{-03}$	$-1.401710\times 10^{-03}$	$4.775809\times 10^{-02}$	$-1.571554\times 10^{-01}$	$3.301702\times 10^{-01}$	$5.735499\times 10^{-01}$	$1.220775\times 10^{+00}$	0.0	$2.234503\times 10^{+00}$
**−30**	$1.726676\times 10^{-04}$	$-1.876306\times 10^{-03}$	$8.591587\times 10^{-03}$	$-2.043407\times 10^{-02}$	$1.991599\times 10^{-02}$	$3.209726\times 10^{-02}$	$-1.707736\times 10^{-01}$	$3.900556\times 10^{-01}$	$4.999761\times 10^{-01}$	$1.154701\times 10^{+00}$	0.0	$2.091379\times 10^{+00}$
**−25**	$2.987991\times 10^{-04}$	$-3.234465\times 10^{-03}$	$1.508602\times 10^{-02}$	$-3.847183\times 10^{-02}$	$5.106311\times 10^{-02}$	$2.115844\times 10^{-03}$	$-1.718213\times 10^{-01}$	$4.468163\times 10^{-01}$	$4.226065\times 10^{-01}$	$1.103378\times 10^{+00}$	0.0	$1.974546\times 10^{+00}$
**−20**	$3.675723\times 10^{-04}$	$-4.117444\times 10^{-03}$	$2.016103\times 10^{-02}$	$-5.536583\times 10^{-02}$	$8.589762\times 10^{-02}$	$-3.968167\times 10^{-02}$	$-1.583298\times 10^{-01}$	$4.976727\times 10^{-01}$	$3.420290\times 10^{-01}$	$1.064178\times 10^{+00}$	0.0	$1.878980\times 10^{+00}$
**−15**	$2.205849\times 10^{-04}$	$-3.164027\times 10^{-03}$	$1.883420\times 10^{-02}$	$-6.106734\times 10^{-02}$	$1.126114\times 10^{-01}$	$-8.611040\times 10^{-02}$	$-1.304906\times 10^{-01}$	$5.401205\times 10^{-01}$	$2.588526\times 10^{-01}$	$1.035276\times 10^{+00}$	0.0	$1.801069\times 10^{+00}$
**−10**	$-3.253779\times 10^{-04}$	$1.176820\times 10^{-03}$	$5.380607\times 10^{-03}$	$-4.356866\times 10^{-02}$	$1.158782\times 10^{-01}$	$-1.260194\times 10^{-01}$	$-9.088799\times 10^{-02}$	$5.721049\times 10^{-01}$	$1.737021\times 10^{-01}$	$1.015426\times 10^{+00}$	0.0	$1.738203\times 10^{+00}$
**−5**	$-1.343520\times 10^{-03}$	$9.612900\times 10^{-03}$	$-2.337590\times 10^{-02}$	$5.496690\times 10^{-03}$	$8.220527\times 10^{-02}$	$-1.469072\times 10^{-01}$	$-4.415609\times 10^{-02}$	$5.921393\times 10^{-01}$	$8.721537\times 10^{-02}$	$1.003819\times 10^{+00}$	0.0	$1.688512\times 10^{+00}$
**0**	$-2.612452\times 10^{-03}$	$2.062429\times 10^{-02}$	$-6.365822\times 10^{-02}$	$8.317309\times 10^{-02}$	$7.741371\times 10^{-03}$	$-1.392950\times 10^{-01}$	$4.005959\times 10^{-03}$	$5.993477\times 10^{-01}$	$4.079083\times 10^{-05}$	$9.999995\times 10^{-01}$	0.0	$1.650697\times 10^{+00}$
**5**	$-3.505380\times 10^{-03}$	$2.956944\times 10^{-02}$	$-1.015639\times 10^{-01}$	$1.688251\times 10^{-01}$	$-9.413551\times 10^{-02}$	$-1.018186\times 10^{-01}$	$4.832874\times 10^{-02}$	$5.934291\times 10^{-01}$	$-8.716225\times 10^{-02}$	$1.003820\times 10^{+00}$	0.0	$1.623931\times 10^{+00}$
**10**	$-3.131190\times 10^{-03}$	$2.950034\times 10^{-02}$	$-1.145989\times 10^{-01}$	$2.243921\times 10^{-01}$	$-1.899840\times 10^{-01}$	$-4.497800\times 10^{-02}$	$8.557826\times 10^{-02}$	$5.745621\times 10^{-01}$	$-1.737226\times 10^{-01}$	$1.015427\times 10^{+00}$	0.0	$1.607800\times 10^{+00}$
**15**	$-8.318476\times 10^{-04}$	$1.451255\times 10^{-02}$	$-8.067694\times 10^{-02}$	$2.062244\times 10^{-01}$	$-2.332728\times 10^{-01}$	$9.209863\times 10^{-03}$	$1.155970\times 10^{-01}$	$5.433080\times 10^{-01}$	$-2.589574\times 10^{-01}$	$1.035278\times 10^{+00}$	0.0	$1.602288\times 10^{+00}$
**20**	$3.116320\times 10^{-03}$	$-1.510245\times 10^{-02}$	$6.392590\times 10^{-03}$	$8.908521\times 10^{-02}$	$-1.838192\times 10^{-01}$	$3.408169\times 10^{-02}$	$1.409622\times 10^{-01}$	$5.005998\times 10^{-01}$	$-3.421779\times 10^{-01}$	$1.064180\times 10^{+00}$	0.0	$1.607797\times 10^{+00}$
**25**	$7.095550\times 10^{-03}$	$-4.897012\times 10^{-02}$	$1.222922\times 10^{-01}$	$-1.059961\times 10^{-01}$	$-3.587599\times 10^{-02}$	$1.196574\times 10^{-02}$	$1.646584\times 10^{-01}$	$4.479184\times 10^{-01}$	$-4.227058\times 10^{-01}$	$1.103379\times 10^{+00}$	0.0	$1.625212\times 10^{+00}$
**30**	$8.663460\times 10^{-03}$	$-6.855341\times 10^{-02}$	$2.115060\times 10^{-01}$	$-2.987652\times 10^{-01}$	$1.587543\times 10^{-01}$	$-4.966493\times 10^{-02}$	$1.860165\times 10^{-01}$	$3.877007\times 10^{-01}$	$-4.999064\times 10^{-01}$	$1.154700\times 10^{+00}$	0.0	$1.656026\times 10^{+00}$
**35**	$6.176410\times 10^{-03}$	$-5.757463\times 10^{-02}$	$2.122160\times 10^{-01}$	$-3.745419\times 10^{-01}$	$2.948737\times 10^{-01}$	$-1.098456\times 10^{-01}$	$1.968569\times 10^{-01}$	$3.238448\times 10^{-01}$	$-5.732360\times 10^{-01}$	$1.220771\times 10^{+00}$	0.0	$1.702549\times 10^{+00}$
**40**	$6.430317\times 10^{-04}$	$-1.716290\times 10^{-02}$	$1.039967\times 10^{-01}$	$-2.565133\times 10^{-01}$	$2.660746\times 10^{-01}$	$-1.084142\times 10^{-01}$	$1.820907\times 10^{-01}$	$2.618004\times 10^{-01}$	$-6.422827\times 10^{-01}$	$1.305402\times 10^{+00}$	0.0	$1.768271\times 10^{+00}$
**45**	$-4.004473\times 10^{-03}$	$2.623103\times 10^{-02}$	$-5.014044\times 10^{-02}$	$1.359082\times 10^{-03}$	$6.281352\times 10^{-02}$	$-1.442622\times 10^{-02}$	$1.308222\times 10^{-01}$	$2.071525\times 10^{-01}$	$-7.067290\times 10^{-01}$	$1.414209\times 10^{+00}$	0.0	$1.858467\times 10^{+00}$
**50**	$-3.511990\times 10^{-03}$	$3.363623\times 10^{-02}$	$-1.149912\times 10^{-01}$	$1.754467\times 10^{-01}$	$-1.341824\times 10^{-01}$	$9.938799\times 10^{-02}$	$6.248129\times 10^{-02}$	$1.610076\times 10^{-01}$	$-7.660815\times 10^{-01}$	$1.555723\times 10^{+00}$	0.0	$1.981283\times 10^{+00}$
**55**	$2.552360\times 10^{-03}$	$-1.333287\times 10^{-02}$	$2.309853\times 10^{-02}$	$-2.179308\times 10^{-02}$	$2.982732\times 10^{-02}$	$4.350840\times 10^{-03}$	$6.294364\times 10^{-02}$	$1.105789\times 10^{-01}$	$-8.188824\times 10^{-01}$	$1.743443\times 10^{+00}$	0.0	$2.149767\times 10^{+00}$
**62**	$9.301310\times 10^{-03}$	$-8.530507\times 10^{-02}$	$3.257453\times 10^{-01}$	$-6.793057\times 10^{-01}$	$8.522229\times 10^{-01}$	$-6.193269\times 10^{-01}$	$3.030676\times 10^{-01}$	$1.579939\times 10^{-02}$	$-8.609945\times 10^{-01}$	$1.999948\times 10^{+00}$	0.0	$2.385939\times 10^{+00}$
